# Development and Validation of an Instrument to Measure Work-Related Stress among Rescue Workers in Traumatic Mass-Casualty Disasters

**DOI:** 10.3390/ijerph18168340

**Published:** 2021-08-06

**Authors:** Yu-Long Chen, Wen-Chii Tzeng, En Chao, Hui-Hsun Chiang

**Affiliations:** 1 Department of Emergency Medicine, Taipei Tzu Chi Hospital, Buddhist Tzu Chi Medical Foundation, New Taipei City 231, Taiwan; yulong0129@tzuchi.com.tw; 2 School of Medicine, Tzu Chi University, Hualien 970, Taiwan; 3 School of Nursing, National Defense Medical Center, Taipei 114, Taiwan; wctzeng@mail.ndmctsgh.edu.tw; 4 Department of Medical Affairs, SongShan branch, Tri-Service General Hospital, Taipei 105, Taiwan; fajohnny@g.ukn.edu.tw

**Keywords:** work-related stress, disaster rescue workers, traumatic events, mass-casualty incidents, disaster management

## Abstract

Rescue workers are a population at high-risk for mental problems as they are exposed to work-related stress from confrontation with traumatic events when responding to a disaster. A reliable measure is needed to assess rescue workers’ work-related stress from their surveillance of a disaster scene to help prevent severe PTSD and depressive symptoms. The purpose of this study was to develop and validate the Work-Related Stress Scale (WRSS) designed to measure stress in rescue workers after responding to traumatic mass-casualty events. An exploratory sequential mixed methods procedure was employed. The qualitative phase of the item generation component involved in-depth interviews of 7 experienced rescue workers from multiple specialties who had taken part in 1 or 2 mass-casualty events: the 2018 Hualien earthquake or the 2016 Tainan earthquake. In the quantitative phase, a modified Delphi approach was used to achieve consensus ratings by the same 7 raters on the items and to assess content validity. Construct validity was determined by confirmatory factor analysis using a broader sample of 293 rescue workers who had taken part in 1 of 2 mass-casualty events: the 2018 Hualien earthquake or the 2021 Hualien train derailment. The final WRSS consists of 16 items total and 4 subscales: Physical Demands, Psychological Response, Environmental Interruption, and Leadership, with aggregated alphas of 0.74–0.88. The WRSS was found to have psychometric integrity as a measure of stress in rescue workers after responding to a disaster.

## 1. Introduction

Traumatic disasters with mass casualties incur major human and healthcare costs in countries such as Taiwan that provide many opportunities for such events to occur [1]. The effects of these catastrophes are experienced not only by the victims at the disaster site; they also create a high risk of long-term negative psychological consequences in the rescue workers [2,3,4]. Rescue workers, especially firefighters, are first responders who provide immediate support to victims at a disaster site. Those who work with multiple other professionals on a rescue team (e.g., in a search and rescue operation) have been found to experience several specific physical–psychosocial–environmental–organizational challenges, such as problems related to food and drink, job-related conflicts with the control tower, lack of cooperation from other team members, confusion about who has what duties, and harsh work environments that require observing and identifying very badly damaged bodies of children and adults [5,6]. Despite these multiple challenges, there are no instruments designed to measure stress surveillance in rescue workers after deployment to traumatic disasters [7].

Rescue workers, especially first responders, are at a high risk for experiencing high levels of psychosocial work stress and depression [8]. In this population, these psychosocial factors appear to be more complex and multifaceted than the environmental factors and the physical and mental strain evoked by work-related accidents [9]. Rescue workers must confront mass-fatality incidents involving many dead bodies and/or body parts as well as emotional involvement with the deceased victims, at times under the threat of injury to themselves [10]. Greater physical strain has been associated with unfair distribution of work tasks, and greater psychological strain has been associated with frequent differences of opinion with other workers or supervisors that interfere with the work [8]. Other research on the stress experienced by rescue workers has shown these stressors to include environmental factors and ineffective cooperation with team members or those in the control tower in the aftermath of a disaster [5]. Direct or indirect exposure to such work environments plays an important role in creating negative psychological consequence in rescue workers when the circumstances are extreme and inhumane and the emotional reactions toward the victims are overwhelming [11].

Work-related stress has been significantly associated with depressive symptoms, burnout, and PTSD symptoms among rescue workers [11,12]. They are at a high risk for physical and mental problems, as they are confronted with traumatic events and endure work-related stress exposure [13]. It is important to develop an instrument for assessing work-related stress specifically for rescue workers who experience physical and psychological impairment after responding to a disaster [14]. The measures of work-related stress currently available have been criticized for the heterogeneous nature of the populations to which they have been applied and their non-specificity re disaster-related events [15,16], especially traumatic mass-casualty events [13]. Our aim was to fill this knowledge gap by developing and evaluating the psychometric integrity of a measure of work-related stress designed specifically for rescue workers.

## 2. Methods and Results

We developed and validated the WRSS across different cultures and types of traumatic mass-casualty disasters, using a succession of qualitative and quantitative methods. In so doing, we followed the recommendations of Zhou [17], who proposed a mixed methods model that features application of both factor analysis and application of mixed-methods integration procedures to culture-relevant data obtained through qualitative interviews to assess the construct validity of a test instrument.

### 2.1. Item Generation for the WRSS

#### 2.1.1. Participant Recruitment and Eligibility Criteria

The original sample for the scale construction consisted of 7 disaster rescue personnel (2 females and 5 males) with various search and rescue (SAR) backgrounds: logistics, search, rescue, SAR dog controller, security officer, and commander. Each had taken part in the response to one of two traumatic mass-casualty events in Hualien, Taiwan: an earthquake on 6 February 2018 or a train derailment on 2 April 2021. The earthquake measured 6.4 on the momentary magnitude scale. It claimed at least 17 dead and 285 injured, and featured bodily mutilations [18]. The train derailment claimed 49 dead, most featuring bodily mutilation or body parts, and a further 247 injured and requiring medical treatment [19]. Both traumatic disasters had the potential to cause members of the SAR team to experience physical–psychological stress at the time and psychological and emotional trauma later [14]. The demographic data for the rescue workers are presented in Appendix A Table A1.

#### 2.1.2. The Interview (Qualitative Component)

Qualitative data were collected through in-depth face-to-face semi-structured interviews of the above 7 rescue workers, all of whom who agreed to be interviewed. The focus of the interviews was first on participants’ experiences of their most recent SAR operation and on how they experienced the work-related stress and the effects of the SAR procedures on them (see Appendix A Table A2). The interviews lasted 45 to 90 min.

The qualitative analysis of the interview data included several steps. In the first step, the researcher analyzed the verbatim transcriptions of the audio-taped interviews using thematic analysis [20]. The transcripts were first read to obtain a general sense of the topic and bring it into focus. There was no sorting or coding of the data in the first step. In the second step the transcripts were reread, and descriptive phrases were extracted. In the third step, the relevant phrases were coded, keeping the codes as similar to the participants’ meanings as possible. In the fourth step, the researcher developed categories from the codes by aggregating similar codes. The codes and categories were examined and compared within and across items to identify relevant relationships. The researcher’s inferences were analyzed and compared with those from the 7 interviewees using a form of peer debriefing.

The four thematic categories of the causes of the traumatic stress identified by the researcher in the third step of the analysis were: physiological demands (of the stress), psychological response (to the stress), leadership (by the team leader), and environmental interruption (at the disaster site). The researcher then generated the 69 original items of the WRSS so they would correspond to these themes.

#### 2.1.3. The Modified Delphi Approach (Quantitative Component) 

The 69 items were further evaluated to achieve consensus from the same 7 rescue workers (raters) using a modified four-stage Delphi method [21]. In this study, the Delphi process required three rounds of item selection and modification. In Round 1, the judges were asked to independently rate each of the 69 statements on whether they agreed that it is a good measure of traumatic stress using a 5-point Likert scale (“strongly agree”, “agree”, “adequate but needs modification”, “disagree”, “strongly disagree”) in the context of responding to a disaster. It has been demonstrated that 5-point scales produce stable findings in modified Delphi studies [22]. The raters were also provided an opportunity to elaborate or explain their ratings and contribute further ideas for each category through responses to open-ended questions provided by the researcher. Using this information, the researcher eliminated 35 items in which there was less than 70 percent of inter-rater agreement and reworded some of the remaining 34. The rescue workers then rated these 34 items in Round 2. The Round 1 process was repeated, which led the researcher to eliminate 6 more items according to the previous criterion. The entire process was repeated in Round 3, leading to the elimination of 9 more items. The ratings of each of the remaining 19 items met the criterion for consensus, defined as >70% of participants affirming the good quality of the item by choosing the response “agree” or “strongly agree”. This definition of consensus has been considered appropriate in previous Delphi studies [23]. Results from the modified Delphi procedure are reported in Appendix A Table A3.

### 2.2. Content Validity of the WRSS

Content validity is the degree to which an instrument covers the conceptual domain of the construct it is intended to measure [24]. The content validity of the 19-item WRSS was assessed by a panel of 3 judges with professional expertise in disaster rescue and emergency management. All were instructors with the Disaster Medical Assistance Team (DMAT) and had personally witnessed several catastrophic disasters in Taiwan. They rated each item on whether it reflected traumatic stress using a 4-point Likert scale with the following response options: “agree very much”, “agree”, “agree a little bit”, and “not agree at all”. The content validity of the scale was found to be 0.94 by using the content validity index (CVI) to detect the validity of the items.

### 2.3. Construct Validity of the WRSS

#### 2.3.1. Participant Recruitment and Eligibility Criteria

Using an online sample-size calculator for structural equation models, it was estimated that 305 participants would be required to detect a moderate effect (*ρ* = 0.23) of high work-related stress for rescue workers who had a high workload [13]. This allowed for 5 latent variables, 19 observed variables, power of 0.8, and alpha of 0.05 [25].

Purposive sampling was used to recruit the participants from March 2018 to May 2021. A sample of 305 rescue workers who were engaged with the 2018 Hualien earthquake or the 2021 Hualien train derailment disasters agreed to participate in the study after the purpose and procedure had been explained by the researchers, who were referred by the occupational nurse. Participants were assured that their participation was anonymous and would not influence their work. Of the 305 rescue workers recruited, 12 were unable to complete the questionnaire because of work-shift rotation problems. Data from the remaining 293 rescue workers were used for the analyses.

#### 2.3.2. Procedure and Data Analysis

After signing the consent form, it took about 15–20 min for the participants to complete the questionnaire in a quiet room in a comfortable environment. The questionnaire consisted of the 19-item WRSS and items requesting the same demographic information obtained from the rescue workers who participated in the 2018 Hualien earthquake or the 2021 Hualien train derailment disasters. We used two separate boxes to collect the consent forms and questionnaires to assure anonymity.

Table 1 presents the demographic characteristics of the sample. To summarize, most participants were men (*n* = 283, 96.6%), were married (*n* = 191, 65.2%), and were between 31–40 years old (*n* = 175, 59.7%). Most reported that their SAR experience was less than or equal to 5 years (*n* = 236, 80.5%), and that their service as firemen was less than 5 years (*n* = 200, 68.3%).

Following the recommendations of Zhou [17], confirmatory factor analysis (CFA) was employed, without a prior exploratory factor analysis, to examine the construct-based validity of the 19-item WRSS using *Mplus* software version 8.2 [26]. The purpose was to confirm a common factor pattern and to determine whether the scale structure in fact corresponds to the four themes identified from the interviews. The determination of which items were to be retained for the final scale was based on their model fit with the factor pattern [27] and conformance to the guiding theoretical definitions of the work-related stress dimensions [2].

Results from the CFA indicated that a four-factor solution was best. After 3 items were removed from the scale because of cross-loadings of the variables, the remaining 16-items fell into four factors (or subscales) with appropriate names corresponding to the four themes derived from the interviews: (1) Environmental Interruption (four items); (2) Psychological Response (five items); (3) Leadership (four items); and (4) Physiological Demands (three items). Participants responded to each item on a four-point scale: (a) “not at all stressful”; (b) “a little bit stressful”; (c) “stressful”; and (d) “very stressful”. Average variance extracted (AVE) was > 0.5 except for Psychological Response (0.43), but the composite reliability for this subscale was higher than the acceptable level of 0.6 [28]. Table 2 presents the CFA results for each item on each factor and Table 3 presents the items on the final scale.

### 2.4. Reliability of the WRSS

The reliability of the 16-item WRSS was assessed through internal consistency analysis.

Cronbach’s alpha values for the four subscales ranged from 0.74 to 0.88 and the composite reliability values ranged from 0.79 to 0.88. For the total scale, Cronbach’s alpha was 0.89 (see Table 2).

### 2.5. Ethical Considerations

In conformance to the ethics requirements, for each phase of scale construction and validation it was emphasized that participants’ cooperation was voluntary and that their answers were confidential and would be used only for the purposes of this study. All participants provided their written informed consent. The Institutional Review Board of the Tri-Service General Hospital approved the study (Approval No. 1-107-05-061).

## 3. Results and Discussion

### 3.1. Main Results

The present study demonstrates that the WRSS has good psychometric integrity. The finding from the interviews that this work-related stress has four dimensions is noteworthy, and four subscales corresponding to these four dimensions (Physical Demands, Psychological Response, Environmental Interruption, and Leadership) were identified in the construct validation phase. We can thus conclude that the WRSS is a well-validated and reliable instrument that is suitable for assessing work-related stress in rescue workers responding to traumatic mass-casualty incidents.

Proper preparation has been shown to be crucial before experiencing the onset of a catastrophic event [13]. Our results indicate that rescue professionals and administrative officers need more specific information about the work-related stress that rescue workers experience and the associated challenges to prepare them to rescue and save the lives of victims. Moreover, in the interviews, the participants registered strong complaints against their administrative officers for not providing them with safety procedures and the necessary control of the environment during SAR events, as well as against the mass media. The findings of our research, which was specifically targeted to disasters, may provide information that will help rescue workers succeed in their rescue operations and improve the quality of disaster SAR. They therefore highlight the importance of developing a specific work-related stress measure for rescue workers.

### 3.2. Comparisons with the Literature

Our results are consistent with what has been found in previous studies that have explored the high physical and mental health risks faced by disaster responders [4,29]. In our interviews, participants expressed a lack of preparation for deployment, specifically a lack of rest and poor sleep quality. These results are consistent with previous research that has shown that rescue workers’ health status, inability to fully regain energy, exhausted from disaster-support work, and perceived physical disturbances play an important role in the creation of work-related stress and aggravate the psychological burdens they face [30,31]. Unpredictable situations involving a threat to human life, such as those faced by disaster rescue workers, have been shown to cause an increase in cardiac sympathetic excitation [32].

High-quality team leadership has been shown to be significantly associated with a lowered risk of subsequent mental distress [33]. Good team leadership plays an important role in employee health and well-being as well as reducing burnout [34]. It is also an essential antecedent of occupational safety; rule-oriented leadership that formulates plans for future operations collaboratively with the workers improves the workers’ motivation to comply with safety regulations and to participate in safety-promoting activities [35]. Our results indicate that rescue professionals and administrative officers need more specific information about the work-related stress that rescue workers experience and the associated challenges in order to prepare them for rescue missions and to save the lives of victims. The results of this study are consistent with those of a previous study demonstrating an increased risk of harmful consequences of simply being in the disaster environment; they include infectious disease, catastrophic injury to oneself or coworkers, severe burns, and psychological stress [36]. These results, as well as our own, call for increased attention to rescue workers’ safety needs and the overall consequences of their work after the deployment.

### 3.3. Strengths and Limitations

Although the results of the present study are a valuable contribution to the literature, several limitations should be noted. Although the instrument has adequate overall psychometric integrity, the number of rescue workers available to serve as raters was small because there are so few catastrophic events that workers respond to. The resulting small sample size in the scale construction phase could have impacted the accuracy of the estimates from the measurement models. Likewise, the samples of raters in both phases may not have been representative of the population of workers who respond to traumatic mass-casualty disasters, because the period of data collection was small (2018 to 2021). Moreover, most of the raters were young men, few of whom were from an organization, and all of them were from just one culture. These factors limit the generalizability of the findings. Future research should use samples from different populations. Finally, previous empirical studies on work-related stress in disaster rescue, including our own study, have been retrospective rather than prospective; future research should include prediction of the consequences of work-related stress in disaster rescue. The strength of employing an exploratory sequential mixed methods approach for constructing the WRSS is that it provided a better understanding of the experiences of the participants because of the inclusion of a qualitative component. The weakness of our application of this approach is the lack of generalizability of the findings.

## 4. Conclusions

In this paper, the construction of a scale measuring work-related stress in rescue workers with good validity and trustworthiness was described. This 16-item, four-factor instrument, for which there is evidence of good content and construct validity, enables a fast, comprehensive, and systematic assessment of the stress suffered by rescue workers from responding to traumatic mass-casualty disasters. The results should increase understanding of rescue workers’ needs and the stresses they experience while responding to traumatic events; paying attention to the outcomes of this research is likely to be important for improving the efficiency and safety of disaster rescue workers. Rescue professionals and administrative officers need to pay more attention to monitoring the work-related stress of rescue workers and the associated challenges to prepare them for SAR and to save the lives of victims.

## Figures and Tables

**Table 1:** Demographics of the rescue workers and differences in their WRSS scores (*n* = 293).

			Work-Related Stress		
	*n*	%	M	SD	*p* ^a^
Age					0.82
≤30	79	27.0	43.22	8.77	
31–40	175	59.7	43.26	8.29	
>40	39	13.3	42.20	13.32	
Gender					0.15
Female	10	3.4	38.78	10.24	
Male	283	96.6	43.26	9.09	
Marital status					0.31
Single	102	34.8	43.87	8.77	
Married	191	65.2	42.71	9.35	
Firefighting service (years)					0.14
≤5	200	68.3	43.29	7.86	
>5	93	31.7	43.70	10.95	
SAR service (years)					0.77
≤5	236	80.5	43.30	8.72	
>5	57	19.5	42.40	10.92	

*Note.* ^a^ from independent *t* test or ANOVA; SAR: search and rescue.

**Table 2:** Summary of confirmatory factor analysis and reliability of Work-Related Stress Scale (*n* = 293).

Construct/Item	Factor Loading (*T* Value)	Cronbach’s Alpha	AVE	Composite Reliability	Bootstrap 95% CI
PHY		0.85	0.66	0.85	
01 Hard to fall asleep because of poor sleep environment	0.84 (32.46)				1.00–1.00
02 Intermittent sleep	0.89 (38.25)				0.90–1.12
04 Enduring sleeplessness	0.70 (19.83)				0.71–0.98
PSY		0.78	0.43	0.79	
10 The victims are my relatives *^a^*	0.56 (11.44)	Deleted			
11 Seeing my team members get injured at work	0.61 (13.95)				1.00–1.00
13 Expressing my condolences to the victims and not allowing myself to be affected by negative emotions	0.70 (18.22)				0.78–1.26
14 Seeing bodily mutilation or severed body parts	0.69 (17.46)				0.95–1.62
16 Touching the dead body unexpectedly	0.73 (19.73)				0.91–1.47
18 Media interview requests	0.53 (10.88)				0.73–1.15
ENV		0.88	0.65	0.88	
21 No one can be searched and rescued *^a^*	0.69 (19.31)	Deleted			
22 Finding all the victims or remains *^a^*	0.71 (20.94)	Deleted			
24 Insufficient manpower	0.75 (24.33)				1.00–1.00
25 Safety of the SAR process not confirmed	0.84 (35.41)				0.97–1.22
26 Worry about the spread of infectious disease at the disaster site	0.83 (33.37)				0.89–1.15
27 Worry about potential harm to rescue workers during disaster rescue	0.80 (30.88)				1.02–1.32
LEAD		0.74	0.54	0.82	
30 Confusing commands or unclear dispatches from the commander	0.46 (8.93)				1.00–1.00
31 Forced to change the SAR route	0.72 (21.04)				0.71–0.96
33 Differences of opinion with team members	0.88 (39.85)				0.89–1.15
34 Chaotic workplace with dysfunctional command system	0.81 (30.16)				0.91–1.17

*Note.* PHY: physical demands; PSY: psychological response; ENV: environmental interruption; LEAD: leadership. Fit indices: χ^2^(98) = 183.84, *p* ≤ 0.001; CFI = 0.96, TLI = 0.95; RMSEA = 0.06; SRMR = 0.05. CI: confidence interval; bootstrap = 1000; range: lower 2.5% to upper 2.5%. The criterion column refers to the raters’ assessments and specifies how many of them classified each item as 1: strongly disagree; 2: disagree; 3: adequate but needs minor rewording; 4: agree; and 5: strongly agree. *n* = number of raters; %: percent of inter-rater agreement, defined as the percent of participants affirming the good quality of the item by choosing the response “agree” or “strongly agree”; *M*: mean rating assigned to each item. *^a^* deleted following confirmatory factor analysis; item 10: cross-loadings of ENV and PSY (MI = 52.47); item 21: cross-loadings of PSY and ENV (MI = 32.25); items 22: cross-loadings of ENV and PSY (MI = 15.65).

**Table 3:** Final items on the Work-Related Stress Scale and ratings from the modified Delphi method.

			Rating						
Subscale	Item #	Item	1	2	3	4	5	%	*M*
PHY	1	Hard to fall asleep because of poor sleep environment	0	0	2	3	2	0.71	4.00
	2	Intermittent sleep	0	0	1	3	3	0.86	4.29
	4	Enduring sleeplessness	0	0	1	3	3	0.86	4.29
PSY	11	Seeing my team members get injured at work	0	0	2	2	3	0.86	4.14
	13	Expressing my condolences to the victims and not allowing myself to be affected by negative emotions	0	0	1	5	1	0.86	4.00
	14	Seeing bodily mutilation or severed body parts	0	0	1	4	2	0.86	4.14
	16	Touching the dead body unexpectedly	0	0	2	3	2	0.71	4.00
	18	Media interview requests	0	0	1	5	1	0.86	3.86
ENV	24	Insufficient manpower	0	0	2	3	2	0.71	4.00
	25	Failure to confirm the safety of the SAR process	0	0	1	4	2	0.86	4.14
	26	Worry about the spread of infectious disease because of poor environment	0	0	2	3	2	0.71	4.00
	27	Worry about potential harm to rescue workers during disaster rescue	0	0	1	3	3	0.86	4.29
LEAD	30	Confused command of unclear dispatch from the commander	0	0	2	3	2	0.71	3.86
	31	Forced to change the SAR route	0	0	1	4	2	0.86	4.14
	33	Differences of opinion with team members	0	0	2	4	1	0.71	3.86
	34	Chaotic workplace with dysfunctional command system	0	0	2	3	2	0.71	4.00

*Note.* ENV: environmental interruption; PSY: psychological response; LEAD: leadership domain; PHY: physical demands; SAR: search and rescue.

## Data Availability

Datasets related to this article can be requested from corresponding author.

## Appendix A

**Table A1:** Demographics of the qualitative interview raters (*n* = 7).

Variable	*n* (%)
Age (years)	
20–30	2 (28.6)
31–40	3 (42.9)
41–50	2 (28.6)
Gender (males) Marital status	5 (71.4)
Single or divorced	1 (14.3)
Married	6 (85.7)
Job specialties	
Logistics	1 (9.1)
Search and rescue	4 (36.4)
SAR dog controller	1 (9.1)
Security officer	3 (27.3)
Commander	2 (18.2)
SAR service (years)	
5–10	1 (14.3)
11–15	3 (42.9)
>15	3 (42.9)
Number of SAR disasters	
1–2	1 (14.3)
3–5	0
6–10	1 (14.3)
>10	5 (45.5)

*Note.* SAR: search and rescue.

**Table A2:** Semi-Structured Interview Guide.

Main Questions	Follow Up Topics If Necessary
What was your role in this disaster rescue?	
Can you please tell me about your most impressive disaster SAR experience?	Can you please tell me about your experience regarding:
	When, where, how, with whom?
	Why is this the most impressive experience for you?
	Can you please tell me about your experience regarding:
	Perceived challenges ahead?
	Feelings or perceptions?
	Subsequent effects?
Can you please tell me what is the most important stressor you noticed in your past disaster rescues	Can you please tell me about your experience regarding:
	When did you start work?
	Influences or impact?
	Present experience?
	Perceived challenges ahead?
	Late effects or impact?
	Specific challenges?
Can you please tell me how you deal with the effects of your experiences of disaster SAR?	Can you please tell me about your experience regarding:
	Influences on physical health?
	Goal achievement?
	Benefit from psychological support?
	Family relationships?
	Social activity?
	Sleep quality?
	Symptom interference?
	Specific challenges?

*Note.* SAR: search and rescue.

**Table A3:** Numbers of remaining items after rounds 1 to 3 of the Delphi procedure.

Factor	Original	Round 1	Round 2	Round 3
Physical Demands	13	7	5	3
Psychological Response	21	9	6	5
Environmental Interruption	20	12	12	7
Leadership	15	6	5	4
Total	69	34	28	19
